# Women's empowerment, children's nutritional status, and the mediating role of household headship structure: Evidence from sub‐Saharan Africa

**DOI:** 10.1111/mcn.13520

**Published:** 2023-04-24

**Authors:** Aaron K. Christian, D. Yaw Atiglo, Michael A. Okyere, Akua Obeng‐Dwamena, Grace S. Marquis, Andrew D. Jones

**Affiliations:** ^1^ Regional Institute for Population Studies (RIPS) University of Ghana Accra Ghana; ^2^ School of Management, Collaborative Innovation Center for Energy Economics and Energy Policy, China Institute for Studies in Energy Policy Xiamen University Xiamen China; ^3^ School of Human Nutrition McGill University Ste Anne de Bellevue Quebec Canada; ^4^ Department of Nutritional Sciences, School of Public Health University of Michigan Ann Arbor Michigan USA

**Keywords:** anaemia, children's nutritional status, household headship, migration, stunting, women's empowerment

## Abstract

We aimed to examine the association between women's empowerment and childhood nutritional status while accounting for the mediating role of household headship structure. Cross‐country, cross‐sectional quantitative data from the most recent Demographic and Health Surveys (2015–2018) were used. Women's empowerment was measured as a composite index of participation in household decision‐making, attitude towards domestic violence, and asset ownership. Childhood nutrition status was measure as anaemia (haemoglobin concentration < 110g/L), stunting (height‐for‐age z‐scorescore <−2) and the co‐occurrence of anaemia and stunting. Applying the Lewbel two‐stage least squares, women's migration status was used as an instrumental variable. We used data on 25,665 woman‐child dyads from eight sub‐Saharan African countries: Burundi (2016), Ethiopia (2016), Guinea (2018), Malawi (2016), Mali (2018), Zimbabwe (2015), Uganda (2016), and Tanzania (2015). The women were in their reproductive ages (15–49 years) and children were under 5 years old. The findings showed that an increase in women's empowerment index reduces children's likelihood of being anaemic and having a co‐occurrence of anaemia and stunting [coeff (SE), −0.114 (0.025) and −0.072 (0.032), respectively]. Specifically, an increase in asset ownership or decision‐making dimensions of empowerment significantly reduces the likelihood of anaemia and the co‐occurrence of anaemia and stunting among children. Children of empowered women from male‐headed households were more likely to be anaemic and be concurrently anaemic and stunted compared to their counterparts whose mothers were from female‐headed households. Interventions designed to improve childhood nutrition through women's empowerment approaches need to consider asset ownership and instrumental agency of women while acknowledging the mediating effect of household headship typology.

## Key messages

- The mediating role of household headship in women's empowerment and childhood nutrition nexus was explored in eight sub‐Saharan African countries.
- Overall, an increase in women's empowerment significantly reduces anaemia and the co‐occurrence of anaemia and stunting in children.
- Specifically, women's autonomy in asset ownership and decision‐making reduces anaemia and the co‐occurrence of anaemia and stunting in children.
- Children of empowered women belonging to male‐headed households had poorer nutritional status than those in households with empowered female heads.

## Graphical abstract

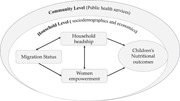

## 1 INTRODUCTION

Women's empowerment (autonomy) is considered the expansion of women's ability to make strategic life choices (Kabeer, 1999). Interest in women's autonomy and empowerment has gained increased attention over the past three decades, not only for its potential effect on women themselves but also on their families, communities, and national development (Zuccala & Horton, 2018). Scholarship, particularly in sub‐Saharan Africa (SSA) links women's empowerment to household and individual outcomes such as health and nutrition (Atiglo et al., 2020; Atiglo & Codjoe, 2019; Onah, 2020; Quisumbing et al., 2021). This is understandable, given that women are the primary caregivers and major gatekeepers in their households when it comes to food and nutrition in SSA (Hatch & Posel, 2018; Mkhwanazi et al., 2018). Findings on the relationship between women's empowerment and child nutrition outcomes are however inconsistent, thus, the need for examination of some nuances in this relationship (Cunningham et al., 2015; Onah, 2020; Santoso et al., 2019).

### 1.1 Women's empowerment and children's nutrition outcomes

**Figure 1:**
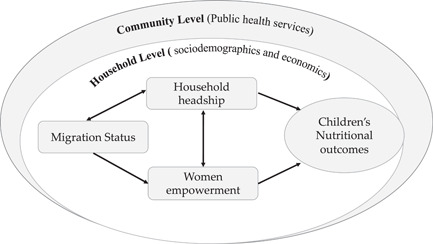
A conceptual framework for the intrarelationships between women's empowerment, household headship and children's nutritional outcomes.

Generally, gendered social and cultural norms influence intrahousehold relationships and are also known to influence child health and nutrition outcomes (Mwaseba & Kaarhus, 2015; Seebens, 2010). Central to research on the relationship between women's empowerment and households' health and nutrition outcomes is the varying interest and expenditure when resources are in the hands of women or men. The inquiry is usually to determine the importance of women's control of resources to the health and well‐being of children who are their primary responsibility when it comes to caregiving. Current scholarship suggests that women with more say or control over resources are more likely to improve the nutrition of their children compared with their counterparts who are less empowered and defer to the will of their husbands or significant others in the household (Gribble & Preston, 1993). Generally, women are more likely than their male counterparts to use household cash to buy food and provide health care for their children (Porter, 2016; UNICEF, 2011), which has a positive impact on household‐level calorie availability and health outcomes. The control of resources by women may reflect positively on household nutrition and well‐being (Santoso et al., 2019), however, women are generally disproportionately found to lack some assets and wealth which may deprive them of the benefits associated with their control of such. Conversely, previous studies have shown household structure, such as headship structure to influence children's nutritional status (Mikalitsa, 2015). However, these studies failed to empirically test how different household headship types may mediate the effect of women's empowerment on childhood nutrition. This study intends to address this gap in the literature by answering the following questions: (i) does women's empowerment affect children's nutrition? (ii) if so, which dimensions of women's empowerment affect children's nutrition? (iii) does the type of household headship mediate the role of women's empowerment on children's nutritional status? Figure 1 is a conceptual framework explaining the intrarelationships between women's empowerment, household headship and children's nutritional outcomes.

In answering the above questions, this study contributes to current scholarship in the following ways. First, we add to the literature on the determinants of childhood malnutrition, particularly the double burden of undernutrition, which is specifically the co‐occurrence of stunting and anaemia. This is because previous investigations on the effect of women's empowerment on children have been limited mostly to the relationship between women's empowerment and single malnutrition conditions (Mikalitsa, 2015). Given the similarity of contributing factors of malnutrition conditions and the extra burden of double malnutrition conditions, current literature argues for the need to examine factors contributing to the presence of double malnutrition conditions in individuals (Christian et al., 2020; Christian et al., 2023; Christian & Dake, 2022). Secondly, we contribute to the empirical literature on empowerment by employing robust estimation techniques to examine specific domains/dimensions of women's empowerment that influence the different measures of childhood malnutrition. Inconsistencies in findings concerning women's autonomy and empowerment have been partly due to the varied differences in WE measurement (Cunningham et al., 2015). This study examines the effect of different dimensions of women's empowerment on childhood nutrition measured as stunting, anaemia and the co‐occurrence of stunting and anaemia.

## 2 METHOD

Our primary source of data was the most recent round of the nationally representative Demographic and Health Survey (DHS) of eight SSA countries—Burundi (2016), Ethiopia (2016), Guinea (2018), Malawi (2016), Mali (2018), Zimbabwe (2015), Uganda (2016), and Tanzania (2015). These were countries that collected data on all variables of interest (migrant status, household headship, women's empowerment, and children's nutritional outcomes). In each country, the prevalence of the double burden of malnutrition is over 30% (33% in Zimbabwe to 58% in Burundi). The DHS is a cross‐sectional, nationally representative health survey conducted in selected low‐ and middle‐income countries approximately every 5 years. Respondent sampling, selection, and administered questionnaires are standardised to make DHS data comparable over time and across countries. The DHS employs a stratified cluster sampling procedure to select census enumeration areas based on a probability proportional to the size of the enumeration area. Households are then randomly sampled within each of the selected enumeration areas. After seeking informed consent from respondents, questionnaires are administered through face‐to‐face interviews with household heads and selected individual household members, including women of reproductive age (15–49 years). The DHS questionnaire included questions on the demographic characteristics of women and their household characteristics. We used household‐level and individual sociodemographic variables and anthropometric measures of children under 5 years. The Ethics Committee of ORC Macro Inc. and the Institutional Review Boards of collaborating institutions in the different study countries provided ethical approval for data collection. All study activities, for example, respondent recruitment and selection and data collection were guided by the 1964 Declaration of Helsinki and its later amendments or comparable ethical standards. No further Institutional Review Board approval was required to access the DHS data as it is deidentified and publicly available.

### 2.1 Measurement of variables

#### 2.1.1 Women's migration status

The DHS captures migration by asking ‘How long have you been living continuously in the current place of residence?’ Respondents who said ‘always’ are treated as ‘non‐migrants’, and those who reported ‘number of years lived in the current place of residence’ are considered as ‘migrants’ if they changed the place of residence across district boundaries.

#### 2.1.2 Household headship

A household is considered a unit or group of individuals living together and sharing similar food and other essential living arrangements (Randall et al., 2015). Households were classified as either male‐headed or female‐headed households. This was based on respondents' answers to the question: ‘Please tell me the name of each person who usually lives here, starting with the head of the household’. The household head is considered an adult male or female who is responsible for the organisation and care of members of the household and is considered the head by members of that household.

#### 2.1.3 Women's empowerment

A broad body of scholarship demonstrates that women's empowerment is linked to their role or ability to partake in important household decisions, their likelihood of suffering from or tolerance of domestic violence and whether they own assets in the family they belong to. In this study we used three main domains of women's empowerment adopted by the standard DHS questionnaire:

1. Women's role in household decision‐making (based on responses to questions relating to women's participation or say in six different types of household decisions on (a) woman's own earning (b) what to do with the money the husband earns, (c) visits to family or relatives, (d) large/major household purchases, (e) daily needs major purchases, and (f) respondent's health care). A composite trichotomous measure was obtained by summing women's responses into low (score of 0), moderate (score of 1–3), and high (score of 4–6) levels of participation in decision‐making.
2. Women's attitude towards domestic violence, as represented by questions asking women whether they agree or disagree with five scenarios in which a husband is justified in beating their wife (when she burns food; argues with her husband; goes out without telling her husband; neglects children; and refuses husband sex). A woman that says ‘NO’ is considered empowered in that domain and given a score of ‘1’. A woman that says ‘YES’ is considered not empowered in that domain and given a score of ‘0’. Their ‘no’ or ‘yes’ responses were summed to form a three‐category variable consisting of low (0), moderate (1–3) and high (4 and 5) autonomy categories.
3. Asset ownership by women, measured by asking women whether they owned a house alone or jointly; owned a land alone or jointly. Their responses were summed to create a variable which was categorised into no (0 positive response), moderate (1 positive response) and complete ownership of assets (2 positive responses).

Categorising the various empowerment indicators into low, moderate, and high has been adopted by several researchers that seek to examine how women's empowerment influences household outcomes (Asim et al., 2022; Rizkianti et al., 2020).

The three individual empowerment domains were utilised in some models in this analysis to assess their differential individual influence on children's nutritional status. A composite variable of women's empowerment was also computed by summing the response categories for the three empowerment domains (participation in household decision‐making, attitude towards domestic violence and asset ownership) to obtain a continuous score (0–12) which was used to assess the influence of the levels of empowerment on children's nutritional status. Higher scores represented higher levels of empowerment. The data included only women who were married or in consensual unions as the questions on women's empowerment in the DHS were only asked to these. Unmarried women were not asked questions on empowerment.

#### 2.1.4 Children's nutritional status

The DHS collected anthropometric data. Children were classified as stunted if height for age *z*‐scores was less than −2.0 (more than 2 standard deviations below the reference median) (de Onis & Branca, 2016). Stunted children are at higher risk for cognitive impairments, reduced academic achievement, and an elevated likelihood of chronic diseases in adulthood (WHO, 2020). With a drop of capillary blood, a HemoCue (Hemocue Inc.) was used to determine the children's haemoglobin concentration. Children with haemoglobin concentration <110 g/L were considered anaemic. Childhood anaemia is caused predominantly by iron deficiency and is linked to numerous negative consequences including fatigue, weakness, and cognitive, impairments, decreased work capacity, increased susceptibility to infections, physical and cognitive developmental delays. The co‐occurrence of anaemia and stunting (CAS) variable was determined by identifying children who had any severity of anaemia concurrently with being classified as stunted.

#### 2.1.5 Other covariates

Based on existing theory, the covariates selected include (1) child characteristics such as age and sex, (2) mother's characteristics including educational status and age, and (3) household characteristics comprising the total number of household members (household size), access to an improved water source and/or an improved sanitation source and wealth. The categorisation of household water and sanitation sources as improved or unimproved is based on WHO/UNICEF classification. Households were classified as using unimproved drinking water sources if their primary source of water came from a river, stream, pond, unprotected spring or well, lake, canal, dam, or irrigation channel and they did not treat the water (such as boiling, using bleach or chlorine, water filtering, solar disinfection, or letting the water stand and settle). Having an improved water source was defined as having access to any one of the following: piped water, public taps, standpipes, tube wells, boreholes, protected dug wells and springs, and rainwater (WHO/UNICEF, 2015). Households with improved sanitation are those with access to a pour‐flush toilet, ventilated improved latrine or a composting toilet or a pit latrine with slab, and those without any of the improved facilities are considered to be using an unimproved facility. Household wealth was generated using assigned asset weights that were generated from a principal components analysis to create standardised asset scores, which were then categorised into terciles (Rutstein et al., 2004).

### 2.2 Econometric framework and strategy

We employed the probit regression model to examine the relationship between women's empowerment and child nutrition, where 1 represents a child with a malnutrition condition (i.e., an anaemic child/a stunted child/a child who is both anaemic and stunted) and 0 otherwise, the generalised form of the model is presented below:

(1) $${\mathrm{Nut}}_{ij}=\alpha +\beta_{1}{\mathrm{WEmpower}}_{kj}+\sum_{k}\beta_{k}X_{kj}+\sum_{i}\beta_{i}\Psi_{ij}+\sum_{j}\beta_{j}\eta_{j}+\varepsilon_{ij},$$

where, ${\mathrm{Nut}}_{ij}$ is the nutritional status of child i in household j; ${\mathrm{WEmpower}}_{kj}$ represents the empowerment index for woman k in household j; $X_{kj}$, $\Psi_{ij}$, and $\eta_{j}$ denotes a vector of characteristics for woman k, child i and household j, respectively; $\varepsilon_{ij}$ signifies the randomly distributed error term; β′s represent the coefficients of the regressors and α denotes the constant term.

Estimates from Equation (1) are likely to suffer from omitted variable bias, which tends to render our estimate endogenous. Thus, potential unobservable covariates are likely to correlate with women's empowerment and child nutrition, which can bias our estimates and alter the generalisation of the results. We argue that women's empowerment will be influenced by women's migration status which will affect their children's nutritional outcomes. Though it is expected that current migration flows will lead to more women remaining in rural or urban communities to take over typical male roles, there has been an increase in female migration over the past two decades (Pickbourn, 2018). Migration provides women with the opportunity to build human, social and capital assets. While literature shows that when women migrate the care for left behind family, particularly children, becomes the responsibility of other remaining women, the fate of care of the children present with migrant women is less discussed (WHO, 2017). To meet Sustainable Development Goal 5 which emphasises the need for gender equality and the empowerment of women (Esquivel & Sweetman, 2016), it is critical to understand pathways through which migration affects women's empowerment and the resulting consequence on household well‐being such as childhood nutritional outcomes. The exclusion constraint here is that migration should only be associated with women's empowerment and not with children's nutrition. The Cragg–Donald weak identification Wald test and Kleibergen–Paaprk Wald *F* statistic are employed to establish the validity of the instrumental variable.

Since migration status, on the other hand, may correlate with children's nutritional outcome via other channels and may not meet the exclusion restriction, we adopt Lewbel's (2012) two‐stage least squares technique to resolve the endogeneity issue. Following Lewbel (2012), the instruments are constructed based on heteroscedasticity in the error terms and can be used in instances where there are no valid instrumental variables (IVs) or together with accurate external IVs (Lewbel, 2012). The method is briefly discussed below:

(2) $$Y_{1}=X^{\prime }\beta +Y_{2}\delta +\mu_{1},Y_{2}=X^{\prime }\alpha +\mu_{2},$$

where $Y_{1}$ represent the outcome variable (child nutrition); $Y_{2}$ denotes the endogenous variable, women's empowerment; $X^{\prime }$ is the vector of explanatory variables while $\mu_{1}$ and $\mu_{2}$ signify the error terms. This technique utilises an identification strategy that relies on information contained in the heteroscedasticity of $\mu_{2}$ in solving an endogeneity problem in the absence of external instruments. This method assumes that $E\left(XX^{\prime }\right)$ is nonsingular, $E\left(X\mu_{1}\right)=E\left(X\mu_{2}\right)=0,\mathrm{Cov}\left(Z,\mu_{1},\mu_{2}\right)=0$, and $\mathrm{Cov}(\mu_{2}^{2})\neq 0$, where Z equals X a subset of the elements of X, then the instruments are estimated as $\left(Z-\bar{Z}\right){\hat{\mu }}_{2}$ where $\bar{Z}$ signifies the mean of Z. The critical assumption under this technique is that there should be no correlation between regressors and heteroscedastic errors. As a further robustness check in addressing endogeneity in nonexperimental datasets, we used the propensity score matching (PSM). In our case, we estimated the average treatment effect (empowered or not) on child nutrition. The average treatment effect is evaluated as follows:

(3) $$\tau \equiv E\left(\theta_{1}-\theta_{0}|\vartheta =1\right),$$

(4) $$\tau =E\left(E\left(\theta_{1}-\theta_{0}|\vartheta =1,p\left(\varpi \right)\right)\right),$$

(5) $$\tau =E\left(E\left(\theta_{1}|\vartheta =1,p\left(\varpi \right)\right)-E\left(\theta_{0}|\vartheta =0,p\left(\varpi \right)\right)|\vartheta =1\right),$$

where *τ* represents the average effect of the treatment, θ denotes our dependent variable while ϑ signifies the dummy variable, which equals 1 if the woman is empowered and 0 otherwise. The vector of pretreatment variables is represented by the control variables and denoted by ϖ. Following this formulation, the propensity score, p(ϖ) is defined as the likelihood for a child with a malnutrition condition (i.e., an anaemic child/a stunted child/a child who is both anaemic and stunted), given the covariates. Following existing literature, we employed multiple matching techniques that encourage the application of such variant matching methods to ensure robustness. These matching algorithms include regression adjustment, nearest‐neighbour, augmented inverse‐probability weighting, and the PSM technique.

As discussed earlier, empowerment can directly and indirectly affect children's nutritional status through various channels. Neglecting this indirect effect usually biases the estimate (MacKinnon et al., 2012) and therefore, we adopted Baron and Kenny's (1986) stepwise regression method to verify the mediation effect (Baron & Kenny, 1986) The model consists of the following three steps:

(6) $$\begin{array}{lll} M_{ij} & = & \Omega_{0}+\Omega_{1}{\mathrm{WEmpower}}_{kj}+\sum_{k}\beta_{k}X_{kj}+\sum_{i}\beta_{i}\Psi_{ij}\\ & & +\sum_{j}\beta_{j}\eta_{j}+\varepsilon_{ij}, \end{array}$$

(7) $$\begin{array}{lll} {\mathrm{Nut}}_{ij} & = & {℧}_{o}+{℧}_{1}{\mathrm{WEmpower}}_{kj}+{℧}_{2}M_{ij}+\sum_{k}\beta_{k}X_{kj}+\sum_{i}\beta_{i}\Psi_{ij}\\ & & +\sum_{j}\beta_{j}\eta_{j}+\varepsilon_{ij}, \end{array}$$

where $M_{i}$ represents the mediating variable. For $M_{i}$ to serve as a channel of influence, it first needs to correlate with women's empowerment as shown in Equation (6) and render empowerment statistically insignificant or reduce its magnitude when included as an additional variable in Equation (7). The reduction in magnitude can be observed when the coefficient of women's empowerment in Equation (7) is compared with Equation (1). The Sobel test, Monte Carlo test, and Delta test are used to validate the mediator. Following the conceptual framework, in Section 2, we used the household headship structure as our mediator.

### 2.3 Ethics of human subject participation

The current study analysed secondary data from the Demographic and Health Survey (DHS). In accordance with the ethics of conducting research with human subjects, voluntary consent to participate in the survey was sought from respondents before being interviewed by fieldworkers who were trained to conduct interviews. IPUMS DHS granted access to the data.

## 3 RESULTS

### 3.1 Background characteristics

The final analytic sample consisted of 25,665 woman–child dyads from eight countries. The highest proportion (43%) of the women had primary education and the vast majority were living in rural areas (81%). There was approximately the same percentage of male (50%) and female (49%) children. The highest percentage (57%) of the children were 1–2 years of age and the lowest percentage (11%) were 4–5 years of age. The overall prevalence of stunting and anaemia were 38% and 62%, respectively. About a quarter of the children were stunted as well as anaemic (25%). More than half of the households had improved water sources (60%) but unimproved sanitation facilities (55%). There are differences in these observations across countries. For instance, the proportion of women with stunted children was highest in Burundi (56%) and lowest in Zimbabwe (27%) while the proportion of with anaemic children was highest in Mali and lowest in Zimbabwe. The proportion of women from female‐headed households was highest in Zimbabwe (11%) and lowest in Ethiopia (3%). Guinea (77%) and Ethiopia (65%) had the highest proportion of women with no formal education while Zimbabwe (1%) and Uganda (11%) had the lowest. Uganda had the highest proportion of migrant women (84%) while Guinea had the least (32%) (Table 1).

**Table 1:** Selected sociodemographic characteristics of households, women, and children in the study sample.

Variables	Total	Burundi	Ethiopia	Guinea	Malawi	Mali	Zimbabwe	Uganda	Tanzania
*Woman characteristics*									
Education									
No formal education	37.6	46.8	65.3	77.2	13.2	74.0	1.4	10.8	20.1
Primary	43.0	42.7	28.0	10.9	66.8	12.3	30.5	60.9	64.8
Secondary	17.5	9.8	4.5	10.4	18.7	12.4	63.0	22.3	14.3
Tertiary	1.9	0.7	2.2	1.6	1.3	1.3	5.1	6.1	0.8
Overall empowerment score	7.0 (6.9, 7.0)	7.6 (7.4, 7.7)	6.9 (6.7, 7.1)	5.0 (4.7, 5.1)	8.4 (8.2, 8.5)	4.1 (4.0, 4.3)	8.3 (8.2, 8.5)	7.3 (7.1, 7.5)	6.2 (6.0, 6.3)
Migrant (= 1, 0 nonmigrant)	47.3	65.9	33.2	31.9	42.6	44.4	73.4	83.5	43.6
*Child characteristics*									
Female (= 1, 0 otherwise)	49.4	49.9	48.6	48.3	50.9	48.6	49.1	48.5	49.1
Diarrhoea (= 1, 0 otherwise)	19.5	29.7	15.9	15.2	25.9	22.1	20.6	25.9	16.2
Stunted	39.0	56.3	40.3	34.8	36.3	29.8	26.5	29.9	35.3
Anaemia	62.0	62.0	60.1	77.5	65.5	85.9	39.9	56.0	62.1
*Household characteristics*									
Female household head (= 1, 0 male**)**	5.8	3.6	2.7	8.5	5.4	4.0	10.8	6.2	8.4
Wealth rank (ref: low/poor)	37.5	29.7	24.9	26.9	43.2	24.0	62.5	28.0	47.6
Medium/middle	34.2	48.0	63.2	29.3	31.3	33.1	1.0	32.2	20.2
High/rich	28.3	22.3	11.9	43.8	25.5	42.9	36.5	39.8	32.2
Household size, #	6.3 (6.2, 6.4)	5.7 (5.6, 5.8)	5.8 (5.7, 5.9)	7.9 (7.6, 8.3)	5.3 (5.2, 5.4)	7.5 (7.1, 7.8)	5.9 (5.7, 6.1)	5.9 (5.7, 6.0)	7.0 (6.7, 7.2)
Number of children under 5 years									
Drinking water									
Improved	63.2	77.0	50.8	71.8	81.3	62.1	67.3	70.5	49.3
Unimproved	36.9	23.0	49.2	28.2	18.7	38.0	32.7	29.5	50.7
Sanitation									
Unimproved	49.8	48.9	90.0	53.5	20.9	44.2	39.2	61.8	29.5
Improved	50.2	51.1	10.0	46.5	79.1	55.8	60.8	38.2	70.5
Place of residence									
Urban	19.6	8.7	11.3	28.8	12.1	20.5	31.2	22.9	28.6
Rural	80.4	91.3	88.7	71.2	87.9	79.5	68.8	77.1	71.5

*Note*: Values represents the percentage or mean (confidence intervals).

### 3.2 Women's empowerment and children's nutritional outcome

Table 2 presents the estimates of the relationship between women's empowerment and children's nutritional outcomes (anaemia, stunting, and CAS) in the eight study countries. We adjusted for various household, women, and child characteristics and used a rural‐urban dummy variable and country‐fixed effects to capture location heterogeneities. In the pooled analysis, women's empowerment was associated with childhood anaemia and CAS but not stunting. The estimated coefficients reveal that the likelihood of a child being anaemic was reduced by 1.4% and 0.4% for the CAS with a one‐unit increase in the women's empowerment score.

**Table 2:** A probit model on the association between women's empowerment and children's nutritional outcomes.

Variables	Anaemia	Stunting	CAS
*Woman characteristics*			
Empowerment score	−0.014***	0.001	−0.004***
	(0.001)	(0.001)	(0.001)
Education (ref: no education)			
Primary	−0.089***	−0.008	−0.039***
	(0.009)	(0.009)	(0.008)
Secondary	−0.146***	−0.074***	−0.091***
	(0.012)	(0.012)	(0.010)
Tertiary	−0.206***	−0.229***	−0.156***
	(0.030)	(0.023)	(0.016)
*Child characteristics*			
Female (ref: male)	−0.024***	−0.062***	−0.047***
	(0.007)	(0.007)	(0.007)
Diarrhoea (ref: none)	0.040***	0.030***	0.038***
	(0.009)	(0.010)	(0.009)
Age (ref: <1 year)			
1–2 years	−0.117***	0.201***	0.112***
	(0.009)	(0.009)	(0.009)
3 years	−0.274***	0.220***	0.073***
	(0.013)	(0.013)	(0.012)
4 years	−0.341***	0.148***	0.021*
	(0.015)	(0.015)	(0.012)
*Household characteristics*			
Wealth rank (ref: low/poor)			
Medium/middle	−0.012	−0.038***	−0.037***
	(0.010)	(0.009)	(0.008)
High/rich	−0.010	−0.093***	−0.075***
	(0.012)	(0.013)	(0.010)
Age of household head (year)	−0.001*	−0.000	−0.000
	(0.000)	(0.000)	(0.000)
Household size, #	0.001	−0.003**	−0.003**
	(0.002)	(0.002)	(0.001)
Total number of children under 5 years, #	0.032***	0.012**	0.018***
	(0.005)	(0.005)	(0.005)
Drinking water (improved) (ref: unimproved)	−0.020**	−0.003	−0.007
	(0.008)	(0.008)	(0.007)
Sanitation (improved) (ref: unimproved)	−0.016*	−0.011	0.008
	(0.008)	(0.009)	(0.008)
Rural (ref: urban)	0.002	0.059***	0.022**
	(0.012)	(0.014)	(0.010)
Country, fixed effect	Yes	Yes	Yes
*F*‐statistics	77.75***	56.79***	49.78***
Mean VIF	3.79	3.80	3.79
Observations	25,647	24,941	25,647

*Note*: Empowerment represents the sum of response categories for the three empowerment domains (participation in household decision‐making, attitude towards domestic violence and assets ownership) to obtain a continuous score (0–12). Wealth tertiles were calculated from an asset‐based wealth index using assigned asset weights from a principal components analysis to create standardised asset scores. Diarrhoea was defined as ‘three or more loose or watery bowel motions in a 24‐h period, as reported by the child's mother or caregiver at any time during the 2 weeks before the interview’ (WHO, 2013).

Standard errors in parentheses.

Abbreviations: CAS, co‐occurrence of anaemia and stunting; VIF, variance inflation factor.

* *p* < 0.1

** *p* < 0.05

*** *p* < 0.01.

### 3.3 Robustness check

Making inferences with these estimates may be problematic as they are likely to suffer from omitted variable bias. Omitted variable bias occurs when the right or appropriate controls are not included or missed in a regression model. Migration was used as an instrument to solve the endogeneity between women's empowerment and child nutrition. The empirical results based on the Lewbel 2SLS regression with both internal and external instruments are presented in Table 3. The Cragg–Donald weak identification Wald test and the Kleibergen–Paaprk Wald *F* statistic suggest that our proposed instrument is valid. In effect, migrant women tended to be more empowered compared to their nonmigrant counterparts and women's empowerment reduced anaemia and CAS among children. These reported results buttress the effect of empowerment on anaemia presented in Table 1.

**Table 3:** A Lewbel 2SLS for the effect of women empowerment on child nutritional status.

Variable	Anaemia	Stunting	CAS
*Panel A—Internal instrument*			
Empowerment	−0.114***	0.020	−0.072**
	(0.025)	(0.022)	(0.032)
Covariates included	Yes	Yes	Yes
*First stage*			
Migration	0.076***	0.078***	0.076***
	(0.010)	(0.010)	(0.010)
Cragg–Donald Wald *F* statistic	59.930	61.383	59.425
Kleibergen–Paaprk Wald *F* statistic	59.684	61.059	59.624
Observations	25,631	24,928	25,631
*Panel B—Internal and external instrument*			
Empowerment	−0.023**	0.010	−0.008
	(0.010)	(0.010)	(0.013)
Covariates included	Yes	Yes	Yes
Cragg–Donald Wald *F* statistic	22.601	22.567	22.601
Kleibergen–Paaprk Wald *F* statistic	12.392	12.491	12.392
Observations	25,631	24,928	25,631

*Note*: Standard errors in parentheses.

Abbreviations: CAS, co‐occurrence of anaemia and stunting; SLS, stage least‐square.

* *p* < 0.1

** *p* < 0.05

*** *p* < 0.01.

As a further confirmatory analysis, we employed the PSM (Table 4) to validate the impact of women's empowerment on child nutrition (anaemia and CAS). Applying the various matching techniques, we observed the average treatment effect on the ‘treated’ to reduce child's malnutrition. Results show that the children of empowered women are about 4.6%–5.9% less likely to be anaemic and 1.4%–2.3% less likely to be anaemic and stunted concurrently.

**Table 4:** Propensity score matching for the effect of women's empowerment on child nutritional status indicators.

Matching technique	ATT for anaemia	ATT for CAS
Nearest‐neighbour matching	−0.046***	−0.014**
	(0.007)	(0.006)
Regression adjustment	−0.059***	−0.023***
	(0.006)	(0.005)
Propensity‐score matching	−0.060***	−0.024***
	(0.008)	(0.007)
Augmented inverse‐probability weighting	−0.059***	−0.023***
	(0.006)	(0.005)
Observations	25,647	25,647

*Note*: Standard errors in parentheses.

Abbreviations: ATT, average effect of treatment; CAS, co‐occurrence of anaemia and stunting.

* *p* < 0.1

** *p* < 0.05

*** *p* < 0.01.

### 3.4 Heterogenous analysis

We tested the effect of the various dimensions of women's empowerment (asset ownership, decision‐making, and domestic violence) on two of the outcomes (anaemia and CAS; Table 5). An increase in asset ownership and the decision‐making dimension of women's empowerment significantly leads to children being less likely to be anaemic (Panel A, Table 5). The effect can be observed to be relatively higher for women with high autonomy.

**Table 5:** A probit model on the effect of the dimensions of women empowerment on childhood nutritional outcome.

Variables	Childhood nutritional outcome					
	Anaemia	Anaemia	Anaemia	CAS	CAS	CAS
Asset ownership (base: none)						
Moderate	−0.115***			−0.074**		
	(0.032)			(0.035)		
Complete	−0.144***			−0.017		
	(0.028)			(0.029)		
Covariates included	Yes			Yes		
Decision‐making (base: none)						
Moderate autonomy		−0.174***			−0.042	
		(0.037)			(0.037)	
High autonomy		−0.382***			−0.128***	
		(0.038)			(0.036)	
Covariates Included		Yes			Yes	
Attitude towards domestic violence (base: low autonomy)						
Moderate autonomy			0.045			0.044
			(0.036)			(0.037)
High autonomy			−0.055			−0.022
			(0.039)			(0.036)
Covariates included			Yes			Yes

*Note*: Standard errors in parentheses.

Covariates included type of household drinking water, household sanitation, number of children under 5 years, sex of child, diarrhoea episode of child, woman's educational status, household location (rural vs. urban) county effects and household size.

Abbreviation: CAS, co‐occurrence of anaemia and stunting.

* *p* < 0.1

** *p* < 0.05

*** *p* < 0.01.

Children whose mothers had moderate and complete ownership of their assets were 11.5% and 14.4% less likely to be anaemic, respectively. A similar finding was observed for CAS. Children whose caregivers/mothers owned moderate levels of assets were about 7.4% less likely to have a double burden of anaemia and stunting, compared to those whose mothers had no assets. Similarly, children whose caregivers/mothers had high decision‐making autonomy were 38.2% and 12.8% less likely to suffer from anaemia and CAS. However, the domestic violence dimension of empowerment had no impact on either outcome.

**Figure 2:**
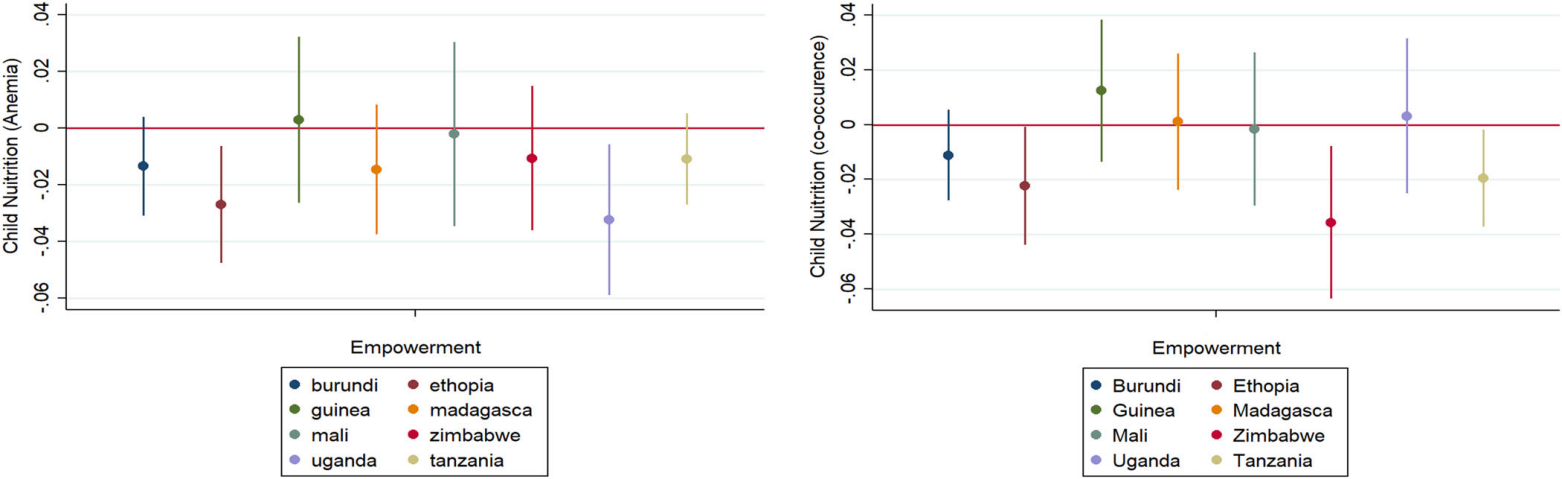
A subgroup analysis of the effect of women empowerment on childhood anaemia (at the left) and CAS (at the right) status by country. CAS, co‐occurrence of anaemia and stunting.

Figure 2 presents a subgroup analysis of the impact of empowerment on anaemia and the CAS among children in eight countries included in this study. Results show that except for Guinea, women's empowerment significantly reduces anaemia in other African countries, with Uganda recording the highest impact. The results for the co‐occurrence of stunting and anaemia present some slight differences. Except for Guinea, Madagascar, and Uganda, women's empowerment significantly reduces the CAS in Burundi, Ethiopia, Mali, Zimbabwe, and Tanzania. The impact is more significant in Zimbabwe compared to the other countries. Therefore, the reduction in the CAS among children of empowered women in Zimbabwe is about twice the effect in Burundi, Ethiopia, and Tanzania.

### 3.5 Channel of influence (empowerment‐household headship‐children's nutrition outcome)

The methods applied in the above analysis accounted for the direct impact of women's empowerment on childhood nutritional outcomes. However, women's empowerment could indirectly affect children's nutritional status through other channels. Table 6 presents an analysis of a possible channel through which women's empowerment could influence child nutrition using Baron & Kenny's (1986) stepwise approach. Women's empowerment was associated with the household structure. The results showed that the household headship structure was significantly associated with anaemia and CAS in children, respectively.

**Table 6:** Mediation analysis with household headship structure as a channel of influence of women's empowerment and children's nutritional status.

Variable	Dependent variable				
	Headship structure	Anaemia	CAS	Anaemia	CAS
Empowerment	0.014***	−0.014***	‐0.006***	−0.012***	‐0.005***
	(0.001)	(0.012)	(0.011)	(0.001)	(0.001)
Headship structure				0.031***	0.014*
Ref: male‐headed households					
				(0.008)	(0.008)
Covariates	Yes	Yes	Yes	Yes	Yes
Delta test (*p*‐value)				0.001	0.073
Sobel test (*p*‐value)				0.001	0.073
Monte Carlo test (*p*‐value)				0.001	0.073
Relative importance testing				0.026	0.040

*Note*: Standard errors in parentheses.

Abbreviation: CAS, co‐occurrence of anaemia and stunting.

* *p* < 0.1

** *p* < 0.05

*** *p* < 0.01.

However, the inclusion of household headship structure as an additional control variable in these models reduced the magnitude of the coefficient of women's empowerment when compared with their corresponding estimates. This showed that household headship structure mediated the relationship between women's empowerment and children's nutrition outcomes. The Sobel, Delta, and Monte Carlo tests further validated this mediator as a channel of influence. The ratio of the indirect effect to the total effect showed that headship structure accounts for about 2.6% and 4.0% of the effect in the relationship between empowerment and anaemia and CAS in children, respectively. With male‐headed households as the reference, the results showed that children in female‐headed households that are empowered are less likely to be anaemic and also less likely to concurrently have anaemia and stunting.

## 4 DISCUSSION

This study is the first to investigate how household headship structure mediates the effect of women's empowerment on childhood anaemia, stunting and the CAS across different SSA countries. The study shows that women's empowerment, particularly in asset ownership and decision‐making autonomy, is protective against childhood nutritional anaemia. This result is consistent with others who have shown that an increase in control of women's mobility and expenditure leads to better nutritional outcomes for their children (Hou, 2016; Shroff et al., 2011). This can be attributed to the fact that the more control women have over resources, the more likely they are to increase expenditure on household expenditures such as food, health, and the general well‐being of their children. These findings corroborate the findings from other SSA contexts showing that domains of empowerment which reflect in asset and instrumental agency are clearly linked to child nutritional status (Jones et al., 2019). Empowerment of women is seen as critical for enhancing infant and young child feeding and household energy availability and dietary diversity in some low‐ and middle‐income countries, contributing to improved diets and nutritional status (Jones et al., 2019; Sraboni et al., 2014). However, given that not all the domains of women's empowerment (specifically domestic violence) influenced childhood nutritional outcomes, women's empowerment should be considered as a multi‐ rather than unidimensional concept. In other words, empowerment in one sphere of life might not necessarily imply a positive effect in other spheres of individual well‐being or household outcomes (Dodoo et al., 2019). Some attempts to address the multidimensionality of the concept of women's empowerment, its measurement, and its impact on household outcomes are realised in the development of the Women Empowerment in Agricultural Index (WEAI) that focuses on agency rather than resources or achievement; agency directly considers the issue of choice or decision‐making (Malapit et al., 2014).

Household headship was found to mediate the effect of women's empowerment on childhood anaemia and CAS. The results demonstrated that, compared to male‐headed households, children in female‐headed households with empowered women are less likely to be anaemic and less likely to simultaneously have anaemia and stunting. Although some previous research has shown that female‐headed households are usually poorer and less food‐secure than households with male heads (Atiglo et al., 2020; Flatø et al., 2017; Tibesigwa & Visser, 2016) the caregiving benefits of female‐headed households that are empowered for their children's wellbeing is being highlighted in this study. This may be explained by the fact that children in such households benefit from the caregiving advantages of belonging to a female‐headed household while also being influenced by the resource pool brought about by an empowered female head. The current finding buttresses the importance of identifying specific pathways or contexts within which women's empowerment influences child nutrition (Sony et al., 2020).

The results show that migration influences children's nutritional outcomes (anaemia and CAS) via women's empowerment. de Brauw et al. (2021) confirmed that women's empowerment was more likely to increase when female household members migrated than when male members of the same household migrated (de Brauw et al., 2021). Despite the possibility of increased their vulnerability to exploitation and abuse, extant research shows that the effect of migration on women is that they are more empowered, although not in all domains.

Migration can be liberating for women and an opportunity for them to escape social restrictions or gender discrimination (Deshingkar & Grimm, 2005; Hugo, 2000). Women can have better access to a variety of education and work options because of migration, which can contribute to reducing income and wealth inequality directly or indirectly (Eryar et al., 2018).

The above findings notwithstanding, the study is not without limitations. DHS surveys have a cross‐sectional nature where data is collected at a single point in time. However, this limits the ability to establish causality or study changes in variables over time. Despite using a complex sampling design to obtain a representative sample of the population, DHS surveys may have some biases in the sample due to nonresponse, under‐representation of certain subgroups, or measurement errors. Furthermore, DHS data are based on self‐reported information, which may be subject to recall bias, social desirability bias, or other measurement errors. Additionally, it is also important to note that measures of women's empowerment are still evolving, with attempts to address both intrinsic and collective agency, as in the use of the WEAI. Regardless, the DHS measures have been thoroughly researched and are widely accepted as addressing important domains of empowerment in the SSA context.

## 5 CONCLUSION AND RECOMMENDATION

This paper investigated the effect of women's empowerment on children's nutritional outcomes, mediated by household headship and women's migration status. Findings reinforce the importance of maternal autonomy and empowerment when targeting interventions to improve the nutrition of children in households. Attention however must be paid to the domains of empowerment that intervention seeks to improve and the household dynamics, particularly the headship structure in which women and children find themselves. Programme managers will have to design interventions to promote women to participate in decision‐making processes, especially those related to health care and nutrition, which can help them make informed decisions that benefit their families. Additionally, there is a need to implement policies that promote gender equality. Given the robustness of our results, we could say that investing in women's empowerment may not only be essential for the well‐being of women but also to ensure better nutrition for their children.

## AUTHOR CONTRIBUTIONS

Aaron K. Christian, D. Yaw Atiglo, Grace S. Marquis, and Andrew D. Jones conceived the study. Aaron K. Christian, Michael A. Okyere, and Akua Obeng‐Dwamena devised the analysis plan. Michael A. Okyere and Akua Obeng‐Dwamena conducted the analyses and interpreted the results. Aaron K. Christian wrote the first draft of the manuscript. All authors read and approved the final version of the manuscript.

## CONFLICT OF INTEREST STATEMENT

The authors declare no conflict of interest.

## Data Availability

Datasets of the Demographic and Health Surveys of the countries analysed in the current study are available on the IPUMS website (https://globalhealth.ipums.org) or measure DHS website (https://dhsprogram.com). The datasets are available for download after formal online registration and submission and a detailed project description.
